# Accelerating Genetic Sensor Development, Scale-up, and Deployment Using Synthetic Biology

**DOI:** 10.34133/bdr.0037

**Published:** 2024-06-25

**Authors:** Shivang Hina-Nilesh Joshi, Christopher Jenkins, David Ulaeto, Thomas E. Gorochowski

**Affiliations:** ^1^ School of Biological Sciences, University of Bristol, Bristol BS8 1TQ, UK.; ^2^ CBR Division, Defence Science and Technology Laboratory, Porton Down, Wiltshire SP4 0JQ, UK.; ^3^ BrisEngBio, School of Chemistry, University of Bristol, Bristol BS8 1TS, UK.

## Abstract

Living cells are exquisitely tuned to sense and respond to changes in their environment. Repurposing these systems to create engineered biosensors has seen growing interest in the field of synthetic biology and provides a foundation for many innovative applications spanning environmental monitoring to improved biobased production. In this review, we present a detailed overview of currently available biosensors and the methods that have supported their development, scale-up, and deployment. We focus on genetic sensors in living cells whose outputs affect gene expression. We find that emerging high-throughput experimental assays and evolutionary approaches combined with advanced bioinformatics and machine learning are establishing pipelines to produce genetic sensors for virtually any small molecule, protein, or nucleic acid. However, more complex sensing tasks based on classifying compositions of many stimuli and the reliable deployment of these systems into real-world settings remain challenges. We suggest that recent advances in our ability to precisely modify nonmodel organisms and the integration of proven control engineering principles (e.g., feedback) into the broader design of genetic sensing systems will be necessary to overcome these hurdles and realize the immense potential of the field.

## Introduction

The ability of all living cells to sense their surroundings and respond to changes in an appropriate way is crucial for their survival. Over billions of years, biological systems have honed their capacity to detect a multitude of environmental features and, based on this information, efficiently regulate gene expression. Examples include ensuring optimal nutrient utilization [1–4], activating appropriate stress responses [5–8], triggering communication between cells [9–11], and controlling the mobility of cells toward valuable resources or away from hazardous environments [12–14]. Typically, a sensor in this context is implemented using a protein or RNA complex that interacts with a target analyte, or biophysical stimulus, to regulate a required cellular response.

From an engineering perspective, sensing is the first step toward control—a long-standing ambition of biological engineering. In this context, the field of synthetic biology, which aims to create biological systems with new and desirable functionalities, strives for predictable, reproducible, and scalable living technologies that can help us better understand biology [15,16] and create novel systems to tackle global challenges in areas like food security [17–19], sustainable manufacturing [20,21], diagnostics [22–25], and healthcare [26–28]. In order to realize this potential, precise control of gene expression is key.

While in principle the development and use of a genetic sensor is simple, the practicalities of repurposing or engineering sensors from scratch that function reliably is incredibly challenging. Blueprints for sensors (i.e., their DNA sequences) are often sourced from organisms that can be difficult to work with. The source organism may grow very slowly [29,30], may be pathogenic [31–34], or may be near impossible to culture under laboratory conditions [29,35,36]. Furthermore, naturally occurring sensors typically lack many of the features necessary for downstream applications. For example, they may have poor sensitivities [37–39], narrow dynamic ranges [40–42], and promiscuous interactions with multiple unwanted targets [39,43–45]. There may also be no known natural sensors for a target of interest, raising the question of where do you start [46–49]? Despite these difficulties, synthetic biologists have developed a range of approaches to discover, create, and systematically modify biosensors for required tasks.

In this review, we cover current trends in genetic sensor design, scale-up, and deployment that have been made possible through advances in synthetic biology and its supporting technologies. We consider a broad range of sensing mechanisms in both whole cells and cell-free systems, with an emphasis on their implementation in microbes. Where possible, we aim to provide the reader with a quantitative understanding of the time, cost, and effort required to create and optimize biosensors using different approaches and the boundaries of our current capabilities in terms of the modalities of sensing and the complexity of the information that can be processed. As synthetic biology solutions move toward real-world applications, embedding advanced sensing and control into them will be essential for realizing their safe and reliable deployment.

## The Anatomy of a Genetic Sensor

**Fig. 1.**
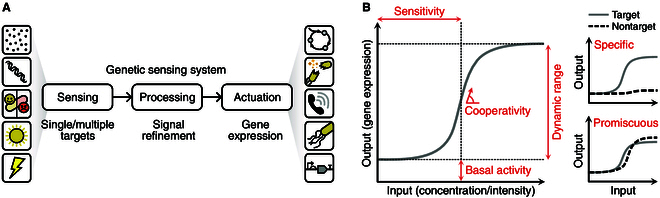
Core elements of a genetic sensor and key features of a sensor response curve. (A) Schematic of a genetic sensing system. While various sensing and processing elements can be combined, changes in gene expression form a common output (actuation) from these systems. Possible inputs include (top to bottom) small molecules, nucleic acids, physiological cell states, light, and electricity, while outputs in gene expression can cause (top to bottom) shifts in metabolism, cell lysis, cell-to-cell communication, motility, and the triggering of more complex gene expression programs. (B) The input–output response of a sensor is crucial for determining its suitability for a specific application. Key features (shown in red) include the sensitivity, dynamic range, basal activity, cooperativity, and specificity/promiscuity of the response.

A genetic sensor consists of 3 core modules. These cover (a) sensing/target interaction, (b) signal processing, and (c) actuation (Fig. 1A). Desirable features of a sensing system include a high specificity for the target, a clear distinction between the OFF and ON states, as well as appropriate sensitivity and cooperativity in response to the target’s concentration or intensity (Fig. 1B). Many of these features can be modified and tuned by optimization of sensor components and the supporting signal processing systems (topics covered later in this review).

In synthetic biology, a major focus of genetic sensor research has been on building diagnostic tools that target small molecules associated with disease states (e.g., metabolites, toxins, and quorum signals) or contaminants (e.g., heavy metals and fertilizer runoff) that may impact the health of humans and the environment. For most diagnostic applications, the output from a genetic sensor is an easy to monitor reporter (e.g., a fluorescent protein or luminescence-producing metabolic pathway) [50]. Point-of-care sensors also often use colorimetric outputs that can be read by eye, such as enzymatic reactions and chromoproteins [23,50], or are interfaced with small electronic devices for digital readouts [24,51]. However, it is important to note that genetic sensors are not limited to merely “reporting” on a target; their output can be exchanged for genes that enable more complex responses. For example, the expression of a biomolecule production pathway [17,52], genetic control networks [53,54], kill switches for biocontainment [55,56], lysogenic genes [26], or the expression of therapeutics [28,57] (Fig. 1A). For these more sophisticated outputs, the sensing system needs to be tightly regulated, as unwanted basal expression could have unpredictable and deleterious effects. Beyond small molecules, genetic sensors have also been created for a myriad of other types of target, including nucleic acids and physical phenomena like light and electricity.

## Discovery of New Genetic Sensors

**Fig. 2.**
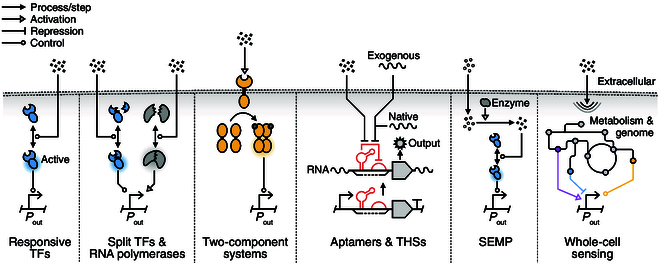
Common types of genetic sensor and their functional mechanisms. Biological systems employ a range of different types of sensor to regulate their behavior. This includes (left to right) target-responsive transcription factors (TFs) that directly regulate the activity of an output promoter; split TFs and RNA polymerases that assemble in the presence or absence of a target to then regulate the activity of an output promoter; two-component systems where a membrane-bound receptor interacts with the target, causing an intracellular signal that regulates the activity of an output promoter; aptamers and toehold switches (THSs) that use RNA–RNA interactions to regulate the translational initiation rate of an output protein; sensing enabled by a metabolic pathway (SEMP), where a synthetic enzymatic pathway is able to convert a target molecule into another molecule that can be more easily sensed and responded to; and whole-cell sensing, where a multitude of intracellular signals (e.g., metabolite concentrations) that respond to a target in a specific way together regulates the activity of an output promoter.

The most common genetic sensors are based on target-responsive transcription factors (Fig. 2). In prokaryotes, these are generally categorized as one-component or two-component systems [58]. One-component systems consist of a protein able to bind its target and directly affect gene expression. In contrast, two-component systems typically consist of a transmembrane-bound sensor histidine kinase protein and a cytosolic transcription factor called the response regulator [59,60]. The sensor histidine kinase typically spans across the membrane, interacting with extracellular targets and transducing this signal to the intracellular histidine kinase domain. This then phosphorylates the receiver domain of the response regulator when activated. However, in some cases, the sensor histidine kinase can also be cytosolic [61] and the phosphorylation/kinase function can be inverted [34,62].

In addition to protein-based sensors, RNA aptamers are also commonly involved in the sensing and regulation of gene expression (Fig. 2). Typically, they are found in the 5′-untranslated region (5′-UTR) of a transcript where they form transcription termination hairpins or complexes that occlude translation initiation sites (e.g., Shine–Dalgarno sequences) in the presence or absence of a target metabolite [63–65].

Single-component transcription factors are the most abundant natural sensors and have been classified into more than 20 major groups [66,67]. Some well-known families include LacI/GalR [68], TetR [66], AraC/XylS [69,70], LysR [71], and LuxR [72], each of which can comprise between 10^3^ and 10^5^ predicted members involved in a broad range of cellular functions covering metabolic regulation, stress responses, antimicrobial resistance, and virulence. These families are often described according to the type of interaction between the protein and its operator sequence (i.e., transcription activating or inhibiting), the relative placement of the operator around a promoter, as well as the type of interaction that the proteins have with themselves. For example, AraC family members typically form a repressive complex in the absence of their target, but transcription-activating complexes in their presence. Many of these sensors can also be identified by structural features, such as helix-turn-helix motifs, which are typical for DNA interactions, and transmembrane-spanning domains in the case of two-component systems.

Most genetic sensors are mined from existing literature, or putative sensors are used to seed a homology-based search in publicly available databases [43,52,73,74]. This method is tried and tested and has been used to discover novel genetic parts, including integrases [75,76], transcriptional repressors [77], alternative sigma factors [78], membrane transporters [79,80], secondary metabolite pathways [81], and CRISPR-Cas systems [82,83]. Homology searches can be carried out using bioinformatics tools such as those available within the National Center for Biotechnology Information (NCBI) database [84], and there are several curated databases for gene regulatory networks, particularly in relation to DNA binding proteins [67,85–89]. However, very few of these focus on genetic sensors [90,91]. In addition to databases, algorithms that employ a “guilt-by-association” strategy have been successfully used to identify potential sensors based on known genomic features like inverted repeat sequences that typically denote operator sites [92–94], or by predicting metabolic pathway products as a proxy for identifying ligand-specific sensors [95,96]. This strategy has also been used to discover RNA aptamers, which are often associated with the 5′-UTR of metabolitic genes [63–65,97].

Machine learning tools have also begun to be applied in this area, in part due to recent advances in protein structure prediction [98–101]. Such tools have been used to identify various aspects of protein-based genetic sensors, including the functional annotation of genetic sequences [102,103], protein–DNA interaction [104,105], and protein–ligand interaction prediction [106–108]. However, these tools struggle to predict the long-range allosteric interactions that play a central role in signal transduction [109]. To overcome this issue, high-quality genotype-to-phenotype data from large experimental screens can be used to support the forward design of genetic sensors [110,111]. This approach was demonstrated by the characterization of more than 60,000 LacI mutants [112] and the use of these data to guide the effective design of novel combinations of mutations that achieve a desired phenotype [110].

Target binding proteins can also be discovered through library-to-library screening, where a curated collection of targets and purified proteins is screened for potential interactions. In these libraries, the ligand and sensor protein candidates are covalently linked to single-stranded DNA (ssDNA) barcodes. If a complex forms between the two, the ssDNA is physically close enough to form a duplex strand that is able to be polymerase chain reaction (PCR)-amplified and read using next-generation sequencing (NGS) [113,114]. In a recent study, a protein candidate library was fused to a terminal deoxynucleotidyl transferase (TdT) that adds a poly-A tail to the ligand’s ssDNA if bound. This signal can then be detected by NGS [115]. Although an effective and high-throughput method for finding protein binding candidates for specific targets, they are limited to molecules that can be covalently fused to ssDNA and the preparation of the libraries is challenging.

For RNA aptamers, given their generally small size (tens of nucleotides), it is possible to create fully randomized 20- to 40-nucleotide (nt) libraries using commercial DNA synthesis. These can then be transcribed and screened in vitro by systematic evolution of ligands using exponential enrichment (SELEX) [116,117]. Randomized 20-nt regions can also be fused to a catalytic RNA domains (ribozymes) [118] to create synthetic aptamers with novel functions (e.g., inducible self-cleaving ribozymes) [116,119,120]. While nucleic acids can engage in functional interactions with ligands, their range of chemical capabilities is limited compared to amino acids and they often exhibit a weak dynamic range, hampering their wider use.

## Sensor Types and Modalities

### Sensing diverse modalities using native promoters

Bacteria have evolved to sense a wide range of intracellular and environmental stimuli. Bioengineers can tap into this natural capability by leveraging native transcriptional regulatory networks to create novel sensing systems. In particular, native *Escherichia coli* promoters have been used for reporting on various physiological stressors. These include chemicals like hydrogen peroxide and nitric oxide [121–123], heavy metal contaminants such as copper, cadmium, magnesium, and zinc [39,124,125], methylating compounds [37], as well as general biomarkers like oxygen [27,126], pH [127,128], temperature [129], and dietary metabolites [123,130,131]. These sensing systems have found applications in diagnosing disease states [122,123], multiplexed sensing for environmental contaminants [39], and the incorporation of kill switches for efficient biocontainment [128,129].

The use of native promoters as sensors has also been used in biobased production to create feedback loops that self-regulate gene expression burden [132,133], for screening functional protein folding [134,135], and for timing auto-induction of gene expression in response to nutrient depletion [74,136] (Fig. 2). Native sensor systems are typically identified using many different methods. These include literature mining [133], bioinformatic analyses [74], and experimental screening using RNA sequencing [132,137].

In addition to using single promoters as sensors, monitoring differential gene expression in response to a stimulus has also been co-opted for genetic sensing. A typical bacterium has thousands of genes involved in maintaining homeostasis in response to environmental fluctuations [4,8,10]. By monitoring global gene expression in response to a target, it is possible to build a predictive model by correlating the input of the target to changes in the activity of hundreds or even thousands of promoters. Advancements in NGS, microfluidics, and machine learning have now made this form of differential sensing possible [138,139].

In one such study [138], a library of 1,807 *E. coli* strains was leveraged, where each had a different native promoter driving expression of a green fluorescent protein (GFP) reporter [140]. Each strain was isolated into a 2,176-chamber microfluidic chip using a spotting robot, and promoter activities were monitored in real-time as media with varying heavy metal ions was washed over the chip. Differential gene activity was then able to be correlated to the heavy metal concentration. This setup proved accurate at predicting contaminant identity and composition in real-world samples. While this platform requires highly specialized equipment and a collection of approximately 1,800 bacteria strains, once established, it is an easy to use and versatile system that can be rapidly adapted for virtually any target.

In a complementary study, transcriptomics was directly used to characterize the promoter activity of approximately 6,000 genes in *Pseudomonas fluorescens* in response to exposure to the pesticide malathion [139]. Although transcriptomics can be expensive, the cost of sequencing has dramatically dropped over the past decade, making this approach feasible for even small laboratories. Furthermore, this method is suitable for any organism of interest and does not require prior host modifications [138]. From the characterized gene responses in the study, a panel of 15 promoters were identified that showed good in silico predictive power. However, when implemented in vivo, the dynamic range of the sensor was 2-fold at best, limiting its potential use.

### Sensing using synthetic metabolic pathways

Small molecules are a common target for many sensing applications, and while targeted mutagenesis and directed evolution can sometimes alter the specificity of a natural sensor partially responsive to a desired target, this approach is not always feasible. To address this issue, scientists have devised alternative strategies that involve constructing synthetic enzymatic pathways designed to convert the target molecule of interest into another type of molecule for which a genetic sensor already exists—an approach known as sensing enabled by a metabolic pathway (SEMP) [141] (Fig. 2). Computational tools such as SensiPath [142] and Galaxy BioSensor [143] have been developed to automate the design process of such systems. These tools have been instrumental in the development of genetic sensors capable of detecting various substances, including cocaine and hippuric acid in urine samples [46], dietary metabolites [143], and pesticides like parathion and atrazine [141,144].

While the SEMP approach is promising for targets that we do not have direct sensors for, it does face several challenges. These include poor sensor dynamics due to competition between synthetic and native pathways for the same metabolic products, difficulties in optimizing multiprotein pathways, and the potential toxicity of the synthetic pathway to the host cell. Many of these issues can be mitigated by the use of cell-free expression systems, where gene expression can be tuned by varying the composition of the cell-free extract [46,144], and metabolite competition and cell toxicity issues are removed. Moreover, it is possible to artificially concentrate these cell-free metabolic systems into protein condensates with densities comparable to those inside living cells [145,146], potentially improving the reaction kinetics and the overall efficiency of the sensing system.

### Sensing nucleic acids

Nucleic acid sensing requires an alternative mechanism to those described for small molecules. Among the various approaches for detecting RNAs, toehold switches (THSs) have proven to be effective (Fig. 2). A THS consists of a synthetic hairpin structure designed into the 5′-UTR of a transcript, blocking the ribosome binding site (RBS) and preventing translation initiation [147,148]. This hairpin sequence can be easily designed using Watson–Crick base pairing and acts as the sensory domain designed to match the target RNA sequence (trigger RNA) [149]. When the trigger RNA is present, it disrupts the hairpin, freeing the RBS for translation initiation. THSs have been used in gene circuit control elements, such as genetic amplifiers [150], basal expression dampeners [151], and multilayered genetic circuit tuning [152,153]. In addition to gene circuits, THSs combined with cell-free systems have significantly impacted point-of-care diagnostics for detecting viral RNA on portable paper platforms [23,154]. The small size and simple kinetics of THSs means that they can be rapidly designed and synthesized at scale [149], with new sensors deployable within a week of identifying a target sequence [154]. Furthermore, THSs have been designed to discriminate targets at a nucleotide resolution, which is essential for sensing rapidly evolving diseases [155]. Many THS applications link the sensor output to easily visualized pigment-producing enzymes, or fluorescent proteins for laboratory-based reporting. Recently, real-time monitoring of THS output has been demonstrated by using restriction enzymes as the sensor output and interfacing this with DNA-coated electrodes [51]. In this reporter system, the restriction enzyme cleaves the DNA attached to the electrodes, altering its conductivity. Such electronic devices have high sensitivity and are compact, making them suitable for use in wide range of environments and applications.

CRISPR-Cas systems have also been adapted for RNA sensing. This involves disrupting the recognition hairpin of the Cas protein-associated single-guide RNA (sgRNA), which can be restored to its full conformation when paired with a target RNA [156–158]. Once complete, the sgRNA-Cas complex is guided to its DNA target recognition sequence independently of the RNA hairpin sequence. In these systems a deactivated Cas protein (dCas) is used for gene inactivation through transcriptional roadblocking [158], gene activation by coupling a transcription-activating domain to a dCas protein [156], as well as DNA base editing, allowing for signal recording in DNA for later retrieval via DNA sequencing [157]. In addition, in vitro CRISPR-based sensing systems have been developed for a broad range of diagnostics [159]. These work via the detection of DNA or RNA of viruses, bacteria, and parasites and have successfully been created for diseases such as *Mycobacterium tuberculosis* [160] and the Epstein–Barr virus [161].

Self-splicing ribozymes have also been repurposed for RNA sensing [162]. This approach involves splitting the ribozyme into two halves that conditionally ligate the two resultant mRNA molecules in the presence of a target RNA. This mechanism has been employed to conditionally unite the halves of a split gene, subsequently activating protein expression. RNA sensors like these have been used to detect transcriptional activity of antimicrobial resistance genes, metabolic pathways, as well as bacterial virulence [156,157,162]. These living RNA sensing systems could serve as valuable tools for in situ monitoring of gene activity within natural microbial communities. By integrating these sensors with bacterial conjugation tools [163,164], genetic programs can be delivered into soil or marine bacteria and then provide direct, on-site reports about specific gene activity within their native environments.

Looking beyond RNA, there has also been recent interest in developing genetic sensors for DNA. DNA is frequently released when cells lyse, and it can remain stable in the extracellular environment for extended periods of time. Several bacteria, including the model bacterium *Bacillus subtilis* [165], are naturally competent, allowing them to readily take up DNA from their surroundings. Researchers have leveraged this ability to develop genetic sensors that can detect a target DNA sequence [25,166,167]. In these systems, a synthetic “landing pad” is created on the cell chromosome, typically consisting of an antibiotic selection marker or a genetic circuit flanked by DNA sequences with homology to the target DNA of interest. When the cell takes up DNA from the environment, the host’s recombination machinery replaces the landing pad with the target DNA only if it is present. This recombination event activates the expression of an alternative antibiotic selection marker or triggers a genetic circuit, reporting the presence of the target. Such DNA sensors have been created to detect pathogenic bacterial DNA from crude fecal samples [166], DNA released by lysed cancer cells in the murine gut [25], as well as for sensing single-nucleotide polymorphisms (SNPs) in human DNA [167].

### Light sensors

Light-based genetic sensors have seen broad interest in synthetic biology because they offer several unique advantages for regulating gene expression. In particular, light activation can be controlled quickly, removed without leaving a trace, and easily patterned, enabling spatially varying gene expression. Genetic light sensors exploit a wide range of mechanisms [168]. These can broadly be categorized into 4 groups: (a) two-component system photoreceptors, (b) one-component proteins that interact with DNA or RNA, (c) light-dependent enzyme reactions, and (d) split proteins that colocalize into a functional component when stimulated by light.

Many genetic light sensors have been transferred from photosynthetic bacteria [169,170] or made synthetically by fusing light interacting domains, such as light-oxygen-voltage (LOV) or bacteriophytochrome domains from plants, fungi, and bacteria, to proteins with gene regulatory functions like two-component systems, DNA binding proteins, and polymerases to achieve light-based activation [168,171,172].

Some of the earliest genetic light sensors were adapted from two-component systems. One of the first was the Cph1 membrane photoreceptor from the cyanobacteria *Synechocystis* fused to the native *E. coli* EnvZ-OmpR two-component system. This enabled red light sensing and was used to demonstrate spatial control of gene expression [173]. Since then, genetic sensors have been created for blue [62,174], green [40,175,176], red [173,175], and far-red wavelengths of light [177–179], and many of these have been used simultaneously in the same cell for multiplexed light sensing [40,180,181].

Synthetic one-component light sensors have also been created, typically by fusing light-interacting LOV domains to the DNA binding domain of LexA [182,183]. In their monomeric state, these proteins are unable to bind to their respective operator sequence. Here, stimulation by light is used to control the dimerization of these monomeric proteins, where light either activates dimerization, resulting in the inhibition of gene expression [182], or alternatively, light is used to disrupt dimerization relieving repression of the inhibitory complex [183]. Recently, light-activated RNA binding proteins have also been created. Notably, a translation inhibition system has been developed using a natural light-activated RNA binding protein that blocks the RBS of an output gene when stimulated by blue light [184]. Complementing control of translation, a synthetic transcription activation system was created by fusing a *B. subtilis* anti-terminator protein LicT to a blue light-activated LOV domain, disrupting transcription termination at the 5′-UTR of a gene of interest in the presence of blue light [185].

Light-activated enzymatic genetic sensors have also been leveraged for gene expression control. These systems produce secondary messengers like cyclic AMP (adenosine monophosphate) or cyclic di-GMP (guanosine monophosphate), which then interact with native regulators to activate gene expression at target promoters [179,186]. However, it should be noted that secondary messengers have global effects on the cell and could cause unwanted impacts on cellular homeostasis.

A fourth type of genetic light sensor uses split proteins that are fused to light interacting domains, similar to the single-component protein systems described earlier. This type of split protein system has been applied to transcription factors [187], T7 RNA polymerase (RNAP) [188–190], and recombinases [191]. In particular, split transcriptional regulators have reversibility, which can be used for dynamic functions such as regulating cell growth rate [192], while recombinase-based systems can be irreversible and have been used to induce mixed genetic populations of antimicrobial resistant cells to study disease [193].

Recently, an additional mechanism for light-based genetic control was reported, which utilizes protein degradation to exert control of gene expression [194]. In this case, the natural conformational change of a LOV domain is used to expose a degradation tag recognized by endogenous proteases, rapidly removing the protein from the cell.

It is important to note that genetic light sensors typically require additional small molecule chromophores for their sensing domains. This often requires the introduction of additional heterologous genes to produce these cofactors, as in red and green light sensors [40,173,195]. Meanwhile, many of the blue light sensors use flavin mononucleotide (FMN) or flavin adenine dinucleotide (FAD), which are readily available in most organisms, making them easier to use in a broad range of hosts [62,171,185,196,197].

The ability for rapid and reversable control using light [189,198] has led to growing interest in using optogenetic control of microbes for therapeutic purposes (e.g., production of beneficial compounds in the gut [57]). For deep tissue applications, genetic sensors for far-red light [169,177,178] are generally more appropriate as this wavelength is the most penetrating through mammalian tissue [199]. To use blue-light sensors for in vivo applications, scientists have created “upconversion” nanomaterials that absorb far-red light and reflect it as blue light [197,200]. These have been used to induce production of a range of therapeutics (e.g., the neurotransmitter GABA, antitumor protein IFN-γ, and neuroprotective protein GCSF) directly in the murine gut using externally applied far-red light. In addition to therapeutics, light-based control can enable spatially patterned gene expression for depositing a bacterial biofilm or pigments on the surface of synthetic materials [201,202], or even in cellulose pellicles to create engineered living materials [21]. Furthermore, the ability for different wavelengths of light to be detected independently allows for multiplexed sensing and has led to the creation of multicolor bacterial photography [203]. Such precise spatial control of gene expression is unique to light genetic sensors and would be near impossible with commonly used chemical inducers.

### Electrogenetic sensors

The prevalence of electronics in modern life has led to growing interest into methods that might interface biology with electrical systems. To do this, the natural redox stress response of bacteria can be harnessed for electrical sensing, where gene expression is dynamically regulated by electrical signals. In these systems, an oxidized species is generated at an electrode (e.g., ferricyanide or hydrogen peroxide), which then interacts with transcription factors involved in oxidative stress to activate gene expression of an output promoter [204–208]. Initial bacterial electrogenetic systems used supplemented redox species, ferrocyanide, and pyocyanin to enable electrical stimulation of the native SoxR protein, which is the master regulator of the oxidative stress response. Using electrodes, researchers were able to demonstrate robust ON/OFF regulation of gene expression that regulated bacterial motility and created two-strain cocultures for signal amplification [204]. Additionally, the distance away from an electrode could be leveraged for additional control, enabling differential gene activity across space [205].

In more recent studies, electrogenetic systems have been developed use hydrogen peroxide generated from oxygen and water at the negative electrode to activate the native OxyR redox regulator [206]. This alternative mechanism avoids supplementing the media with toxic redox species (ferri/ferrocyanide) or antimicrobials (pyocyanin) and can be used in aerobic conditions. Additionally, hydrogen peroxide is a universally recognized molecule, opening the potential to adapt this sensing mechanism to other bacteria using native parts. Researchers have demonstrated control of bacterial coculture growth rate in bioreactors [207], electrostimulated production of amino acids [208], and showed the potential for two-way interaction with electrodes [206]. This opens up the opportunity for engineered cells to be used in compact bioelectric devices where gene expression can be dynamically controlled by altering an external electrical field. In addition to being space-efficient, electrical interfaces enable a means for constant measurements potentially allowing for near real-time observations (e.g., dynamics on the timescale of minutes) [209]. This is an improvement over many optical approaches, where readouts such as fluorescence or colorimetry require tens of minutes for protein expression and maturation before a sufficient signal is detectable.

## Optimization of Genetic Sensors

For a genetic sensor to be useful, it often needs to be optimized for a specific task. This may include the transfer of the sensor into a more genetically tractable host, as well optimization of the response to a target using a systematic design process, or even randomised mutagenic approaches.

### Porting sensors to new hosts

The systematic transfer of genetic parts from one organism to another is often referred to as “porting.” In the context of genetic sensors, a sensor is typically ported from its original host to a model organism such that it is then easier to work with. Common model organisms include *E. coli* (gram-negative) or *B. subtilis* (gram-positive) for bacteria, or *Saccharomyces cerevisiae* for microbial eukaryotes [165,210,211]. Porting removes sensors from their native context, where additional layers of control may dictate the level and timing of sensor gene expression [6,140,212]. Common reasons for developing a sensor outside the native host include a limited understanding of the organisms biology, a lack of techniques for genetic manipulation or cultivation in laboratory conditions [29,30,35,36], and pathogenicity of the host [31–34].

When porting a genetic sensor, there may be some initial nonspecific interactions with the new host’s genome or metabolism [213], issues with expression of the sensor [93], intrinsic autoregulation of sensor components [175], and the need for additional (but unavailable) factors for sensor function [195,214]. Despite these potential issues, model organisms are well characterized with regards to their genetic and metabolic background [86,87,215,216], and there is a wealth of information available on the effects of heterologous gene expression on host physiology [2,3,217,218] to guide improvements. Additionally, model organism have large collections of genetic parts that can be used to fine-tune expression to refine sensor performance [219–221], and DNA synthesis can address incompatibilities (e.g., differing codon usage) and unwanted regulation by refactoring and harmonizing genetic components for the new host [17,222].

### Tuning expression of sensor components

**Fig. 3.**
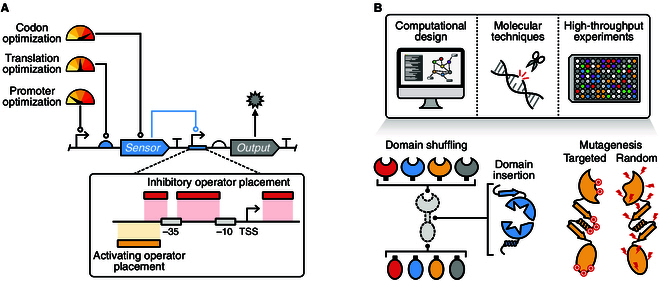
Approaches to optimize genetic sensors. (A) Response characteristics of protein-based sensors can be tuned by varying the expression of the regulator’s transcription and translation, and via modifications to the output promoter structure to alter the inhibition or activation of transcription. (B) Protein engineering of the sensor is becoming possible through emerging computational design approaches, precise molecular techniques to edit DNA, and high-throughput experiments to test libraries of new variants. Modifications can include shuffling of modular domains, insertion of new functional domains, and mutagenesis of the entire, or regions, of the sensor to explore the sensor design space.

Ensuring components of a sensor are appropriately expressed (i.e., in the correct stoichiometries) is crucial for their function. Parts to modulate constitutive gene expression include promoters and RBSs [219] to tune the transcription and translation of the sensor genes, respectively (Fig. 3A). Other essential parts include transcriptional terminators [223,224] and translational insulators [219,225]. The former plays a vital role in preventing nonspecific activation of genes by transcriptional read-through into nearby genes, and the latter ensures that 5′-UTR sequence variation and mRNA secondary structures have a lesser impact on translation initiation rate [225]. In addition to transcription and translation, plasmid copy number can also be used to control gene expression [131,226–230] as most sensing systems are implemented on multicopy plasmids to simplify the prototyping of new genetic devices.

Depending on a sensor’s regulatory mechanism, under- or overexpression of specific components can have a myriad of effects on performance. For example, underexpression of an inhibitory regulator can lead to higher basal expression of the output in the absence of the target, while overexpression can result in insensitivity [214,231,232]. In contrast, the inverse is often true for activating regulators where underexpression leads to sensor insensitivity and weaker maximal activation, while overexpression causes high basal expression of the output [175,233]. Additionally, overexpression of either type of sensor can cause retroactivity [234,235] and burden on the host cell due to excessive expression of heterologous genes [217,236–238]. To address these issues, computational simulations and design of experiment (DoE) frameworks can be used to optimally tune the various sensor parameters [239,240].

When a sensor consists of multiple genes (e.g., two-component systems), or when membrane transporters or additional cofactors are needed [170,173,214,241], optimizing the expression of multiple components at the same time can be laborious and time consuming [195]. An alternative approach is to use chemically inducible promoters to easily tune the expression of each sensor gene [52,175,230,232,242–244]. However, for large multicomponent systems, empirically testing every expression level might still not be feasible. In these cases, mathematical modeling can help guide the design process [214,239,240,245].

Even with these tools, tuning the expression of many sensor genes in living cells is time consuming. An alternative approach is to remove the use of cells entirely and move to cell-free systems. In this context, gene expression can be tuned simply by controlling the concentrations of DNA carrying the genetic component [46,246,247] and removing the need to modify regulatory parts and potentially deleterious impacts on cell growth. Cell-free systems can also be supplied with linear DNA [248,249], such as PCR amplicons or synthetically synthesized DNA fragments, further reducing the reliance on bacteria for producing genetic material for high-throughput characterization. Although cell-free systems allow for rapid prototyping of new genetic sensors, they are hindered by significant batch-to-batch variation in protein expression capacity, which often requires bespoke formulations of salts, nucleoside triphosphates (NTPs), and cofactors to regain functionality [250]. As cell-free systems gain popularity in engineering biology, commercially available reagents meeting high-quality control standards are becoming available [251], albeit at premium price. Furthermore, innovative tools and techniques are emerging from the broader cell-free synthetic biology community, which improve the consistency of “homebrew” cell-free extracts. These advancements include the utilization of alternative culture methods [252], engineering strains to be more optimal cell-free extracts [249], as well as developing machine learning pipelines to rapidly optimize energy mixes for maximum production [253].

### Promoter engineering

In addition to refactoring the regulatory components of a genetic sensor, the output promoter can also be modified to optimize sensor performance. In some cases, the output promoter of a sensor can have additional elements that hinder sensor function [175,254]. These issues are mitigated by modifying the promoter to contain only essential components, as well as refactoring the wider sensing system to remove any unwanted regulation. Promoters in bacteria are fairly well understood [255], allowing synthetic promoters to be created by altering specific regions (i.e., the −10 and −35 sequences) and the placement of operator sequences [233,256,257]. For inhibitory transcription factors, the operators can be placed downstream of, flanking, or overlapping the −10 and/or the −35 core promoter motifs. In contrast, activating transcription factor operators are typically placed upstream at the 5′-end of the core promoter region. Additionally, computational tools have been developed for the automated design of promoters with desired activities [258,259]. Given the short lengths of promoter parts (35 to 200 base pairs), libraries of designs can also be commercially synthesized for efficient cloning and high-throughput characterization [126,260].

### Enhancing sensor function through protein fusions

Sometimes when designing a genetic sensor, additional functionalities are required. Protein chimeras allow us to combine features of multiple proteins into one. Proteins of the same family typically have greater success when using this strategy as structural similarities reduce misfolding at fusion junctions, allowing signals to be transduced from the sensory to actuation domains. This fusion method has been demonstrated extensively for proteins from the LacI/GalR family, where synthetic fusions of ligand binding domains and DNA binding domains have enabled customisable multi-input, single-output gene expression control [261–264].

The highly specialized domains of two-component systems, with their inherent modularity, are well suited implementing new sensing capabilities through this approach [60] (Fig. 3B). Fusion proteins from this class of sensors have been used to create novel optogenetic tools for spatial control of gene expression [62,173,177], as well as enable engineered probiotics to sense and respond to pathogen quorum signals in the murine gut [34]. In all these cases, ensuring signal transduction at domain junctions in these protein fusions is crucial, often necessitating extensive screening of multiple fusion sites to find an optimal variant [125,265].

In addition to enabling new functionalities, fusion proteins have also been used to improve sensor compatibility between hosts. For example, the heavy metal sensory domains of CueR (copper sensor) and ZntR (zinc sensor) from gram-negative *E. coli* were fused to the DNA binding domain of a MerR transcription factor to enable promoter activation in the gram-positive *B. subtilis*—a host better suited for heavy metal sensing [266]. Additionally, response regulators from two-component systems are also ideally suited for protein fusions, where introducing heterologous DNA binding domains to native two-component system response regulators can reduce cross-reactivity and increases dynamic range [267].

As previously mentioned, proteins of similar classes or families are amenable to domain cross-compatibilities due to their structural similarities, allowing for effective signal transmission across the protein. More extreme approaches have also been trialed, where completely different functional domains are fused together to create more radically diverse sensors. These fusions typically either rely on a static mechanism for sensor activation (e.g., colocalization of proteins in the presence of the target) or can also induce conformational changes when interacting with the target.

For static mechanisms, several studies have used native DNA binding proteins as scaffolds onto which a target binding protein is appended. For example, a caffeine binding variable heavy domain of heavy chain (VHH) camelid nanobody was fused to the *E. coli* LexA DNA binding domain to create a caffeine biosensor [48]. Similarly, photodimerization domains have been fused with the LexA protein to achieve light-activated gene expression [182,183]. In both these cases, the LexA domain alone lacks strong DNA binding, so the target binding domain is used to induce a protein complex, allowing it to repress gene expression at a target promoter. Besides gene inhibition, ligand-interacting domains can also be connected to transcriptional activators. For instance, the enzyme idi-isomerase was fused to AraC to create a biosensor for isoprenoid production [268]. This dimerization mechanism can also be applied to create transmembrane sensors, as demonstrated by the fusion of an extracellular bile acid binding domain from *Vibrio cholerae* with the CadC transmembrane protein of *E. coli*, leading to activation of gene expression in vivo in response to bile acid [269]. Additionally, various dimerization interfaces for protein–protein interactions can be used to improve other sensor properties, including increased cooperativity, reduced basal activity, and improved specificity [270–272].

The versatility of simply fusing proteins together has been extended to eukaryotic genetic sensors. Typically, these are composed of a prokaryotic DNA binding protein fused to a transcription-activating domain like the yeast GAL4 or viral VP16 proteins, and multiple copies of the cognate operator DNA sequence being placed at the 5′-end of the gene to be expressed [37,273–275]. Alternatively, target binding proteins can be fused to separate DNA-binding and transcription-activating domains to create artificial heteromeric complexes. These then colocalize in the presence of the target, activating gene expression [21,275]. Additionally, artificial zinc finger domains have also been used as DNA binding proteins with highly tunable binding kinetics [276] to create orthogonal genetic sensors in yeast [277].

Apart from sequentially fusing proteins either at the N- or C-terminus, functional domains can also be inserted in the middle of proteins (Fig. 3B). This is used to create synthetic split protein genetic sensors or enable proteins with novel conformational changes in response to a target of interest. For these kinds of development, there are several examples where Mu-transposition has been used to effectively sample insertion sites within a protein of interest [278,279]. In particular, split sites discovered in T7 RNAP have been leveraged to create transcription-activating genetic sensors by linking target binding domains to each half of the split polymerase. This mechanism has been used to create genetic sensors for various targets including the anticancer drug rapamycin, human hormone estradiol and plant hormone abscisic acid [280,281], as well as blue light by fusing LOV domains to each terminus [189,190]. In addition to T7 RNAP, extracytoplasmic sigma factors (ECFs) are also promising candidates for this form of split transcription activator mechanism, where a caffeine sensing system was created by fusing caffeine binding VHH camelid nanobodies to a split ECF protein [279].

Domain insertions that incorporate new conformational changes have also had some notable success. For instance, an estradiol binding domain has been inserted into dCas9 to enable conditional transcriptional control [282], as well as in the fluorescent protein mCherry to indicate successful binding [279]. However, this latter mechanism is generally more challenging to achieve because of the reliance on protein structures needing to be compatible for successful signal transduction, resulting in most possible designs being nonfunctional or poorly performing at best [283]. This is a bottleneck that could be overcome by the innovation occurring in the computational protein design space, where new protein structures could be engineered to facilitate more effective conformational change at these synthetic domain junctions.

### Mutagenesis and directed evolution

A major challenge in protein engineering is the incomprehensibly vast design space [284]. Structural knowledge can expedite design by narrowing down which regions are optimal for mutagenesis. However, this information is not available for most proteins, and dynamic conformational changes cannot be engineered from structure alone. To address sensor design challenges, randomized mutagenesis and directed evolution strategies strike a balance between effectively sampling the vast protein design space, while also verifying sensor function (Fig. 3B). In this context, a sensor of interest undergoes iterative cycles of sequence diversification (i.e., in vitro or in vivo mutagenesis) followed by function characterization. At the end of each cycle, improved mutants are propagated for further rounds of mutation until functional improvements reach a plateau. Through these strategies, many facets of sensor function can be modified including target specificity [47,49,285,286], sensitivity [41,43,112,287], dynamic range [42,52,288], cooperativity [112], DNA interaction type [38,262,264,289], and stability [290,291].

Mutations can have a broad range of effects on phenotype, and modifications in specific functional domains can alter sensitivities or even target recognition entirely. For example, mutations have been introduced in ligand binding pockets to both improve target specificity [111,292] or even change the target molecule entirely [47,49,285]. Similarly, mutations can be introduced to DNA binding domains to tune operator binding kinetics, as well as create orthogonal operator sequences to increase the multiplexing potential of a limited repertoire of parts [49,262]. In addition to ligand–protein and DNA–protein interaction, protein–protein interfaces can be mutated to alter reactions kinetics (i.e., for histidine kinase domain of two-component systems) [287] as well as improve protein interaction orthogonality [125].

In addition to functional domains, distal mutations can alter protein stability and conformation, and play a major role in sensor function, affecting sensitivity to the target molecule [42,52,288,293], specificity to the ligand [47,285,286], and dynamic range [42,52,288]. Interestingly, distal mutations in inhibitory transcription factors have even been shown to invert a sensor's response [38,112,289]. Of these general mutations, distal modifications are the most difficult to predict as these can have global effects over the entire structure.

Although many proteins may not have experimentally determined structural data, sequence homology to proteins with known structures can be instrumental in identifying key functional features, such as ligand interaction pockets or DNA-binding domains that could be targeted for mutagenesis [43,49,285]. Homology-based searches are simple and fast to execute but lack in precisely predicting the role of sequence and function. More advanced structure determination tools, such as the biophysical modeling Rosetta design suite [47,294,295], can provide deeper insights into protein structure prediction and forward design of genetic sensors, but can be costly in terms of the time and compute required. Complementing biophysical modeling, machine learning pipelines are increasing in popularity for de novo protein structure prediction [98–101] and can be used to elucidate key features [104–108]. To enable more accurate sequence-function predictions, methods combing mutagenesis, NGS, and high-throughput function characterization are being developed, which can generate the information needed to train machine learning algorithms [110,111,296].

### In vitro mutagenesis methods

Saturated mutagenesis by PCR is commonly used to introduce mutations at specific residues of interest [43,49,125,262]. Alternatively, chip-based DNA synthesis can be used to chemically synthesize thousands of variants that can be cloned in one-pot reactions [47,111,126]. For global nonspecific mutagenesis, error-prone PCR is popular due to its simplicity and the ability to tune mutation rates by varying the type of error-prone DNA polymerase, using excess salt concentrations (e.g., magnesium and manganese), or by unbalancing deoxyribonucleoside triphosphate (dNTP) ratios [41,42,112]. Both targeted and global mutagenesis methods can be combined sequentially to further diversify genetic sensors, where saturation mutagenesis is initially used to radically alter sensor dynamics or target specificity. However, this often results in poorly performing sensors, which can then be gradually improved by iterative rounds of global mutagenesis [49,52,286]. In subsequent rounds of mutagenesis, library shuffling is also commonly used to combine multiple beneficial mutations into one sequence [297], and it can even reveal epistatic effects where mutations in isolation have no effect on function, but when combined can drastically improve performance.

Of particular note is an in vitro mutagenesis method called compartmentalized partnered replication (CPR) [52,298,399]. In this method, the sensor output is connected to the expression of an error-prone DNA polymerase, where after induction the cells are isolated in oil emulsion droplets mixed with PCR reagents and primers targeting the evolving gene. These droplets are directly PCR-amplified in a thermal cycler, where the amplification of the template gene will correlate with the level of DNA polymerase expression during the induction phase. This method removes several steps, such as overnight growth of cells and DNA extractions, significantly reducing the cycle time for each round of directed evolution. CPR has been used successfully to evolve genetic sensors for reduced background expression, improved sensitivity, and better orthogonality when distinguishing molecules of highly similar structures [52,298].

### In vivo mutagenesis methods

The advantage of applying mutations directly in vivo is that it avoids iterative rounds of DNA extraction, in vitro mutagenesis, and the need to transform cells. This makes these approaches more amenable to automation, allowing a more thorough sampling of sequence design space [299–301]. In vivo directed mutagenesis is a vast research area [302]. Here, we focus on methods that introduce randomized mutations within the targeted genes. These strategies use various mechanisms, including bacteriophages [280,281,303], orthogonal replication machinery [304,305], and guided mutagenesis [306–308].

Phage-assisted continuous evolution (PACE) [303] has been a pioneering method for in vivo directed evolution and used to evolve orthogonal gene regulators [309], improve antimicrobial production [310], perform enzyme evolution [281], create novel protospacer-adjacent motif (PAM) extended CRISPR proteins and base editors [301,311], and evolve noncanonical amino acid incorporation into proteins [300]. PACE links a genetic sensor’s output to the expression of an essential M13 bacteriophage gene pIII [280,303]. The evolving gene is placed on a defective M13 genome lacking pIII, while the sensor-controlled copy of pIII exists on an accessory plasmid maintained within the *E. coli* host. In a chemostat-based “lagoon,” the phage particles infect fresh *E. coli* cells and are exposed to chemical mutagens and the target of interest. The mutagens genetically diversify the population, and if beneficial mutations accumulate in the sensor gene, pIII expression improves, enabling complete and fully functional phage particles to be produced. The use of a chemostat ensures a constant influx of fresh cells and removal of waste products. This favors the rapid replication of phage particles, which correlates with improved sensor variants. PACE primarily relies on pIII expression for identifying improved designs, but can also incorporate additional reporters like luminescence for visual screening [300]. It should be noted that mutagenesis in this approach is applied globally to the cell, which could have deleterious effects on the selection genes and result in successful mutants being lost. Additionally, the phage life cycle is very sensitive to a multitude of factors, including temperature, salinity, trace metals, and strain specificity, limiting the contexts in which it can be used.

More recently, an in vivo mutagenesis strategy called OrthoRep has also been established, which uses an orthogonal DNA polymerase that only replicates its target plasmid and does not interfere with the host’s native genome [304]. The orthogonal polymerase has been mutated to be highly error prone, resulting in 100,000-fold higher mutation rates in the target plasmid in comparison to the host genome. However, OrthoRep has been primarily implemented in yeast, where it has been used to evolve genetic sensors for cis,cis-muconic acid and adipic acid [312], enzyme evolution [313], as well as being expanded for automated control [299]. Only very recently was a bacterial OrthoRep system developed for *Bacillus thuringiensis* [305] using a similar error-prone DNA polymerase targeting a specific plasmid’s replication. In this case, up to 6,700-fold higher mutation rates are achieved for the plasmid over the background genome mutation rate of the cell. However, a limitation of the approach is that mutations can accumulate in essential components of the mutating plasmid, limiting the length of time that an OrthoRep system remains functional.

More targeted approaches have been developed, which involve the fusion of DNA-interacting proteins and DNA-modifying proteins. In a system called MutaT7, the evolving gene is placed downstream of a T7 promoter and is mutated as the gene is transcribed by a fusion protein of T7 RNAP and a cytidine deaminase [306,307]. Crucially, mutations are specific to the genes being transcribed. The main limitation of this system is that the mutations introduced are limited to C-to-T and G-to-A. In an alternative system, a fusion protein comprising an error-prone DNA polymerase I and nCas9 nickase is engineered to generate mutations at precise locations targeted by a guide RNA [308]. The nCas9-induced DNA nick facilitates DNA synthesis initiation and strand displacement of the wild-type gene, resulting in mutations adjacent to the target site at 7.7 × 10^6^ fold higher rate than background mutations, albeit within a limited region along the DNA.

### Effective sensor screening and selection

Regardless of the mutagenesis method of choice, assessing the vast numbers of variants produced is a major bottleneck, as most mutations will not be beneficial [47,112]. For such libraries, an effective screening or selection method is essential [38,42,43,125]. Screening methods assess every variant within a library for functionality, and those with improved performance are identified and taken forward for further study. In these methods, the output is typically a fluorescent protein, and a plate reader or fluorescence-activated cell sorting (FACS) is used for medium- to high-throughput characterization and isolation of functional variants [183,269,282,292]. A major benefit of FACS is that the sorting is automated and can be carried out in multiple rounds of positive and negative selection. Once functional variants have been successfully isolated, NGS can be used to recover the genotypes of the selected designs [125,162].

Unfortunately, screening mechanisms alone are restrictive due to the majority of the variants being rejected [41,43,112]. FACS instruments can help sort large libraries, but sorting rates often need to be lowered to below 10^3^ events per second to ensure a high accuracy [111,314]. This means that sorting a large library of 10^5^–10^6^ variants could take anywhere from several hours to several days. Furthermore, these instruments are expensive to buy and are high maintenance, which further restricts accessibility of this approach.

Selection mechanisms offer a complementary strategy to fluorescence-based screening where survival of the cell is coupled to the desired function of the genetic sensor. This can be done by simply placing an antibiotic resistance gene as a positive selection marker under the control of the candidate sensor [41,112]. In more versatile selection schemes, the sensor output is also paired with a conditionally toxic gene for negative selection [43,52,286]. In these dual-selection methods, the accurate activation of the sensor is coupled with the expression of a positive selection marker, while the absence of the target is associated with the repression of a negative selection marker. This process helps to eliminate nonfunctional variants from the pool via cell death. Selection mechanisms also offer better discrimination between low levels of gene expression that would be difficult to resolve using fluorescence measurements alone [47].

Common conditional toxin genes used for negative selection include SacB, a gene encoding levansucrase that makes gram-negative bacteria susceptible to sucrose toxicity [315]; RpsL, a dominant streptomycin susceptible allele of an essential ribosomal protein [316]; TolC, a membrane transporter that makes cells susceptible to antimicrobial colistin E1 [47,317]; and PheS, a mutant of the phenylalanine aminoacyl tRNA synthetase that can mischarge the tRNA with the noncanonical amino acid 4-chloro-dl-phenylalanine (Cl-Phe) [52]. Some genes can act simultaneously as positive and negative selection markers such as the membrane transporter TetA [315,318], which confers resistance to the antibiotic tetracycline and also makes the cell susceptible to nickel chloride toxicity.

Dual-selection schemes are able to accurately isolate variants with desired features. However, each round of selection usually requires growth on solid media for 12 to 15 hours, extending the time it takes to perform a complete cycle. To address this issue, researchers have created a synthetic fusion protein by combining the kanamycin resistance gene with the herpes simplex virus thymidine kinase (HsvTK) [274,319]. This forms a protein complex that can rapidly introduce an artificial nucleoside known as dP [6-(β-d-2-deoxyribofuranosyl)-3,4-dihydro-8*H*-pyrimido(oxazin-7-one)] into replicating DNA, which rapidly causes cell death. This accelerated selection mechanism allows for a complete cycle of mutagenesis, transformation, negative selection, and positive selection to be performed within an 8-hour time frame, as opposed to the typical 2 days when using the classical approach [38,42]. Moreover, this selection scheme allows all steps to be carried out in liquid cultures, making it well suited for automation.

As discussed earlier, CPR represents an emerging strategy with a unique selection criterion [52,320,321]. In this approach, a DNA polymerase serves as the positive marker, where the amplification of the template gene in subsequent PCR steps directly correlates with levels of DNA polymerase expressed during the positive selection phase. As previously mentioned, CPR eliminates the need for overnight growth steps and DNA extractions, making it ideal for iterative applications in liquid cultures.

It is often important to adjust selection strategies dynamically because overly stringent criteria can eliminate promising variants, particularly in initial rounds of mutagenesis [43,112,286]. Adjusting the selection mechanism often involves an empirical process of trial and error. This means that libraries may need to be tested several times to tune the stringency of an experiment. In contrast, tuning the screening process for FACS is relatively straightforward, as it can be varied in real time by adjusting gating settings on the machine. It is important to note that screening and selection are not mutually exclusive processes. In fact, they can complement each other effectively. Selection can initially eliminate the majority of nonfunctional mutants, and subsequently, microplate readers or FACS can be employed to screen the enriched collection for further processing [38,41–43,52].

## Expanding the Scope of Sensing Systems via Genetic Circuits

Earlier we showed how components of a sensor can be directly optimized. An alternative approach that offers a means to create more complex sensing systems is to integrate genetic circuitry that can process a sensing module’s activity and shape the output dynamics of the overall system in a desired way. To achieve this, synthetic genetic circuits are often used [322,323]. Notable examples include layered genetic amplifiers that significantly expand the detectable limits of sensor systems by connecting the output of a weak sensor to a cascade of transcriptional activating proteins to enhance and tune reporter expression [324,325]. Alternatively, antisense transcription has been employed to reduce basal activity and improve dynamic range [326,327]. Invertor circuits, constructed by linking the sensor output to a transcriptional repressor, have been used to flip a sensor response and often results in enhanced dynamic range [34,62]. Furthermore, novel protease systems have been used both as actuators for dual activation-inhibition functions [328] and programmable protein removal [136,329,330]. In more elaborate regulatory motifs, the incoming sensor can be coupled to both activating and inhibiting components, thereby creating an incoherent feed forward loop (iFFL). This ensures that output activity is only high for a limited range of input concentrations/intensities [331]. Alternatively, a sensor can be connected to dual activators in a feed-forward loop to create sensor responses with binary switch-like activation responses [151]. A major benefit of this approach is that genetic circuits can be easily pieced together to achieve more advanced functionalities. Of particular interest for sensing systems are the ability to implement memory and complex multi-input logic, which expand the scope of sensing applications.

### Incorporating memory

There is growing interest in the application of genetic sensors as diagnostics tools for the human gut due to their inherent biocompatibility [24,25,73,121,122,127,332–334]. A major challenge in this context is that the diagnostic needs to traverse the length of the intestines and so will be exposed to varying stimuli as it moves along the tract. Most genetic sensors rely on their input being continually present for expression of an output. This makes them inherently incompatible for use in dynamic environments like the human gut.

**Fig. 4.**
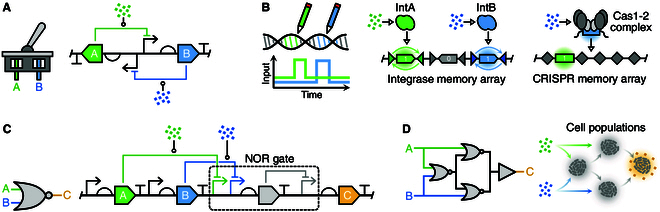
Genetic circuitry for advanced sensing system. (A) Genetic toggle switches generate a bistable system where only gene A or B is expressed at any point in time. Switching between stable states can occur through addition of external molecules to affect the regulation of one of the states, or via overexpression of the desired output protein. (B) DNA can be used as a permanent, nonreversable, memory store using integrases to alter the orientation of specific segments of DNA, or CRISPR-Cas systems to append short chunks of DNA encoding the current state at a point in time. These systems can allow for the presence of a signal to be recovered, even if it no longer persists in the environment. This is useful for signals that are transient in nature. (C) Decision making can be implemented using genetic logic gates to inegrate and process single or multiple sensor inputs. (D) Complex decision making that would be challenging to implement in single cells can be distributed across multiple types of cell population to reduce the cellular burden and enable easily reconfigurable logic.

This problem has inspired researchers to explore the coupling of genetic sensors to memory devices to “record” events over time. Genetic toggle switches were one of the first synthetic genetic circuits and have the capacity to store information [335] (Fig. 4A). A toggle switch consists of two genes that are under the mutual repression of the each other. This creates a bistable system where it is possible for only one of the two genes to be active at a specific point in time. Toggle switches have been coupled to bacterial quorum molecules [336], biomarkers of gut inflammation such as tetrathionate [337] and haem [241,337,338], as well as native bacterial sensors that respond to inflammation [339]. This simple mutually repressing architecture has been expanded by incorporating genetic inverters to create latches that resemble digital computer memory able to process transient pulse-like signals [340]. By carefully characterizing each component in these systems, it is possible to build layers of genetic regulators able to detect varying intensities of a target input [332]. Despite their flexibility, dynamic memory circuits like the toggle switch are prone to errors that cause unwanted switching between states [336,338]. This can be caused by DNA copy fluctuations [226,341], retroactivity [234], or gene expression burden [217] and results in the loss of any stored information.

To complement these dynamic information storage circuits, DNA-modifying circuits have also been built by coupling genetic sensors to integrases [75,121,122,342,343] (Fig. 4B). Integrases are enzymes capable of excising and reorientating DNA at specific target locations governed by recognition sites in a potentially nonreversible way (i.e., enable permanent memories to be stored in DNA). Integrase-based circuits have been used to create band-pass filters for hydrogen peroxide sensing [121] and real-time nitric oxide reporter systems that interface with electronic components [122], used as actuators to multi-input logic circuits [75], and applied in the human commensal gut microbe *Bacteroides thetaiotamicron* to respond to the sugar rhamnose [343].

CRISPR-Cas systems are inherently DNA storage mechanisms that capture foreign DNA, typically from bacteriophages, and integrate portions of this into target arrays in the genome [344] (Fig. 4B). To take advantage of this innate ability for DNA recording, scientists have created genetic sensing systems that can simulate invasion by foreign DNA by coupling the replication of a plasmid to native sensor promoters so that when a target molecule is present the plasmid is rapidly amplified within the cell [130]. This amplification of plasmid DNA is captured by the CRISPR-Cas system and stored in genomic arrays that can be later read by NGS. This type of memory storage has been adapted to respond to a trehalose (a dietary metabolite), fucose (a gut infection-associated biormarker), copper (heavy metal contaminant), as well as electrical stimulation [130,345]. An advantage of CRISPR-based recorders is that they can record multiple events over time as DNA acquisition is a continuous process.

### Multi-input logic for information processing

Some sensing applications require that more than one type of target to be present, or that some combination of target signals are present and/or absent in order for the output to be triggered. To implement this logic, it is necessary for biological computations that can process several inputs simultaneously in order to make a decision. To achieve this, researchers have adapted algorithms from electrical engineering to create tools like Cello [53] that automate the design of genetic logic circuits (Fig. 4C). Cello has been used to create dozens of multi-input genetic circuits in bacteria and yeast [53,346]. It has also been expanded for applications in a prominent gut probiotic chassis strain, *B. thetaiotamicron*, to develop genetic devices able to sense and respond to the gut biomarker deoxycholic acid [333]. While tools like Cello are easy to use, the circuits produced are large and complex, making it difficult to understand the source of failure when they do not work as expected. This can stem from numerous failure modes such as cryptic regulatory elements, unwanted antisense transcription, inefficient transcriptional termination [258,347,348], and burden-induced failure due to the demands of the circuit on the host cell [217,349,350].

One way to overcome such burden is to implement a division of labor where functional elements of a circuit are split between different types of cells that can communicate using quorum sensing molecules (Fig. 4D). This approach has been used to create modular multi-input gene circuits [39], signal amplifiers [245,351], and coordinate gene expression across a population for functions like the delivery of therapeutics to tumors in response to specific biomarkers of pH and oxygen levels [26,229].

## Deployment of Genetic Sensors

Laboratory *E. coli* strains such as MG1655, BW25113, and BL21 are commonly used as hosts for developing genetic sensors, but are not suitable chassis for many real-world applications. In accordance with its long history as a pathogenic microbe [352], many *E. coli* strains carry genes for naturally occurring genotoxins that could have negative effects on humans and therefore do not have “Generally Regarded as Safe” (GRAS) status. In contrast, for nearly a century, the human isolated *E. coli* strain, Nissle 1917, has been used as a commercially available probiotic [353]. Given its long historical use, Nissle 1917 has been the central chassis for several therapeutic projects focusing on gastrointestinal disease and cancer treatment [26,27,314,354–356]. Very recently, a genetically modified version of this strain created by SynLogic to treat phenylketonuria (PKU) has received Orphan Drug Designation by the US Food and Drug Administration, providing a legal precedent for an approved genetically engineered living therapeutic.

Many of the tools for engineering laboratory *E. coli* strains can be readily adapted for Nissle 1917, and there is continued development of novel parts and plasmid tools taking into account the context in which the device is to function. For example, identifying promoters with predictable activities [332,357], novel plasmid tools for the stable maintenance of genetic constructs [334,358], or integrating landing pads directly into the genome for easy genomic manipulation [359], as well as conducting comparative studies of sensors in physiologically relevant conditions (i.e., aerobic versus anaerobic culture) [127].

Researchers have built and deployed numerous genetic sensors in Nissle 1917 for targeting biomarkers unique to the mammalian gut and tumors, including sugars, amino acids, caffeine, temperature, pH, hypoxia, heme, neurotransmitters, and calprotectin [24,73,127,137,229,232]. In addition to endogenous chemical cues, *E. coli* probiotics are also being developed that can respond to physical cues, including light stimulation [57,200] and temperature [129,360]. Interestingly, temperature-based genetic sensors can also be activated by the application of ultrasound to precisely heat localized regions of tissues, such as around tumors, and initiate antibody synthesis [28]. However, Nissle 1917 is often criticized as a poor colonizer of the mammalian gut, restricting its use to only short-term therapeutic solutions. Scientists have demonstrated that *E. coli* strains isolated directly from the murine gut are amenable to heterologous gene modifications [338,361] and can function as long-term genetic sensors in the murine gut on timescales from months to years [337,339]. Given this success, a promising avenue may be to isolate native human *E. coli* as long-term therapeutic hosts.

Apart from *E. coli*, *B. thetaiotamicron* and *Lactococcus lactis* show promise as platforms for human therapeutics. *B. thetaiotamicron* has been engineered with genetic sensors capable of recognizing dietary biomarkers, such as sugars [343,362] and bile acid [333]. These sensors are linked to CRISPRi-based logic gates, allowing them to generate specific responses to multiple inputs. Furthermore, *L. lactis* has been modified to detect *V. cholerae* in the murine gut [34], to produce a secretable split β-lactamase when triggered by xylose to protect the gut microbiome from antibiotic-induced dysbiosis [363], and engineered to produce therapeutic human proteins upon far-red light stimulation from outside the body [197,200].

In addition to diagnostics linked directly to the gut, biosensors designed for environmental monitoring of compounds that can indirectly affect health have also been developed. These cover a broad range of compounds including endocrine disruptors [364] to heavy metals [365].

**Fig. 5.**
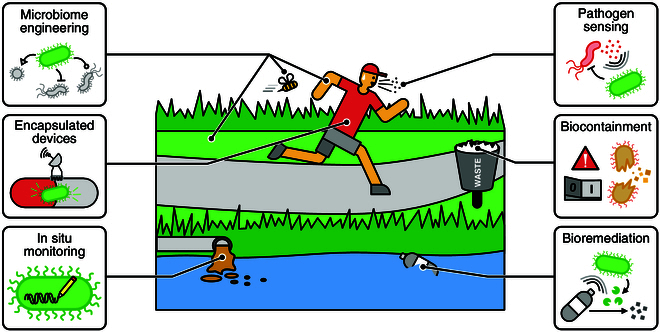
Deployment of genetic sensors for real-world applications. Genetic sensors offer the potential to underpin many key applications to improve our everyday lives. This includes (top to bottom, left to right) sensing and manipulating microbial populations to improve ecosystems (e.g., rhizosphere composition for improved crop yields) and animal/human health (e.g., gut and skin microbiomes); cells containing genetic sensors can be encapsulated to enable long-term monitoring in harsh environments (e.g., digestive tract); the ability to couple genetic sensors to DNA-based recorders enables in situ monitoring of environments; genetic sensors can be developed to respond to molecules produced by pathogens and potentially act to eradicate them; safety is crucial for deployed genetic sensors, and various biocontainment procedures can be used to ensure that engineered biology can be removed on demand; and genetic sensors can be coupled to bioremediation pathways in cells to trigger the recycling of waste on demand.

When using genetic sensors in living cells for real-world applications, it is important to consider biocontainment requirements to prevent engineered organisms from proliferating uncontrollably in the environment (Fig. 5). A simple method to achieve this is encapsulation of cells in hydrogels beads [123,366,367] or housing them in electronic devices [24,122] to physically constrain their movement.

Physical containment is not possible for long-term colonization applications in the gut or in tumors. In these contexts, biocontainment strategies include creating auxotrophic strains [27] and placing essential genes under the control of sensors that restrict engineered cells to specific locations. For example, cells have been engineered to be confined to the low pH and low oxygen environment of tumors [128,229], or in the sugar-rich and 37 ^∘^C warm temperature of the human gut [55,56,129]. Efficiency and long-term stability are crucial for biocontainment kill switches, where some of the most effective devices employ nucleases as actuators, leaving no trace of their DNA after activation [55,221,368].

In addition to endogenous therapeutics, smart genetically modified organisms are also being embedded into engineered living materials (ELMs), such as biofilm-based inks [369,370] and bacteriocellulose-based materials [371], with the intention of incorporating these into new wearable diagnostics [372]. ELMs are also being applied in materials where the regenerative ability of biology is being harnessed to activate repair mechanisms in response to damage [20,373], or using light-based control to pattern pigment production and biomaterial deposition onto the surface of synthetic materials [201,202].

Real-world environments often require us to engineer nonmodel organisms, which remains a significant challenge. Organizations like SEVA (Standard European Vector Architecture) [374,375] initiated some of the earliest efforts in this area by providing open-access genetic parts that work with nonmodel gram-negative bacteria. SEVA used these tools themselves to demonstrate their versatility by creating a wide array of sensors for application in metabolic engineering and bioremediation using *Pseudomonas putida* as a host [242]. Building on of this work, others have created genetic toolkits of various sensors adapted from *E. coli* and ported them to a range of gram-negative bacteria [376–378]. Genetic assembly toolkits have also been developed for diverse bacteria, including the bacteriocellulose-producing *Komagataeibacter rhaeticus* [379], the fast-growing marine bacterium *Vibrio natriegens* [380,381] (a promising chassis for water bioremediation [196]), and even for genetic engineering bacteria from the bee gut microbiome [382] (Fig. 5). It is likely only a matter of time until we will be in daily contact with genetic sensors in our medication, clothing, and food.

## Future Directions

As we have shown, genetic sensor research is a burgeoning field covering a diverse range of targets and sensing mechanisms. While progress in the laboratory has been swift, developing systems that are able to be deployed for wide-scale use remains a challenge due to regulatory constraints. The use of NGS to uncover innate responses of microbes may offer a route forward. NGS techniques are beginning to be used to identify native promoters within diverse microbial hosts that respond to a target of interest [137,139,217]. Given the stringent regulations surrounding genetically modified organisms for real-world use, it would be advantageous to leverage the inherent capabilities of organisms already present where a system needs to be deployed. This strategy gives these technologies the classification of genetically edited organisms (no introduction of permanent heterologous DNA), which have been accepted for field use [19]. With more time and continued progress in employing genetically edited organisms, regulatory bodies and public perceptions may accept the use of these technologies more broadly.

Beyond sensing systems that are readily available in nature, innovative methods using synthetic metabolic pathways are likely to further expand the chemical space that can be detected by genetic sensing systems [141,144]. Similarly, the continued development of nucleic acid strand displacement circuits has expanded our ability to sense specific RNAs for endogenous monitoring of gene activity [147,157,162], and in combination with cell-free approaches, these systems are being deployed for monitoring and tracking rapidly evolving diseases at the nucleotide level [154,155]. More broadly, emerging machine learning based tools like AlphaFold [99] offer immense opportunities to design protein sensors from scratch, avoiding the need to laboriously search and screen natural biodiversity, as well as supporting the creation of sensors for any target molecule (even those new to nature).

Most genetic sensors to date have targeted chemicals in a diagnostic context. Looking forward, it is likely that expanding the remit of genetic sensors to cover biophysical properties of an environment will grow in importance. Existing light and electric genetic sensors already allow us to interface biology with compact electronic and optical devices enabling rapid, dynamic, and reversible control that would be impossible with chemicals alone [189,198]. Exploring avenues for new types of genetic sensor that can monitor broader physical phenomena like pressure [383], magnetism, and gravity are exciting avenues to still be explored.

Another emerging area in this field is the use of sensing arrays for the classification of complex targets where the relative composition of elements is important. These systems often rely on the identification of components with variable sensitives, broad ligand binding capabilities (i.e., high promiscuity), as well as variable dynamic ranges. By exploiting a large set of sensors simultaneously that are able to interact differentially with a range of targets [384], it is possible to rapidly create novel sensors for single targets, or compositions of targets, similar to how natural olfactory systems work.

Looking beyond living cells, the encapsulation of cell-free gene expression systems into lipid vesicles and other membrane-bound compartments has enabled the creation of synthetic cell-based biosensors [385,386]. These have the advantage of allowing for more precisely defined compositions of the molecular parts that are present and offer the ability to rapidly tune the performance of the sensing system present. They also strip away much of the complexity present in living cells, offering routes to more predictive model-based design.

As synthetic biology begins to enter the real world [387], we also need to ensure that we trust the biological systems built. Deploying engineered organisms into our food, water, clothing, and medication requires that we are sure that they will function as expected. Meeting this requirement is challenging due to the complexity of biology. However, fields such as control engineering already address this need in other areas of engineering, using feedback to ensure systems function within a desired operating range. The implementation of control processes using synthetic biology are already emerging [341,388,389], and further integration of control engineering principles into the design of genetic sensors is likely to be an important step to realizing the robustness required for widespread deployment of genetic sensors into our everyday life.
